# Health-related Quality of Life among hospitalized older people awaiting residential aged care

**DOI:** 10.1186/1477-7525-7-71

**Published:** 2009-07-26

**Authors:** Lynne C Giles, Graeme Hawthorne, Maria Crotty

**Affiliations:** 1 Department of Rehabilitation and Aged Care, Flinders University, GPO Box 2100, Adelaide, South Australia 5001; 2 Department of Psychiatry, Faculty of Medicine, Dentistry and Health Sciences, The University of Melbourne, Parkville, Melbourne, Victoria 3050, Australia

## Abstract

**Background:**

Health related quality of life (HRQoL) in very late life is not well understood. The aim of the present study was to assess HRQoL and health outcomes at four months follow-up in a group of older people awaiting transfer to residential aged care.

**Methods:**

Secondary analysis of data from a randomized controlled trial conducted in three public hospitals in Adelaide. A total of 320 patients in hospital beds awaiting a residential aged care bed participated. Outcome measurements included HRQoL (Assessment of Quality of Life; AQoL), functional level (Modified Barthel Index), hospital readmission rates, survival, and place of residence at four months follow-up.

**Results:**

In this frail group the median AQoL was poor at baseline (median 0.02; 95%CI -0.01 – 0.04) and at follow-up (0.05; 95%CI 0.03 – 0.06). On leaving hospital, more than one third of participants who were moving for the first time into nursing home care rated themselves in a state worse than death (AQoL ≤ 0.0). Poor HRQoL at discharge from hospital (AQoL ≤ 0.0) was a significant predictor of mortality (HR 1.7; 95%CI 1.2 – 2.7), but not hospital readmission nor place of residence at four months follow-up. Improved function was a predictor of improved HRQoL among the surviving cohort.

**Conclusion:**

People making the transition to residential aged care from hospital have very poor HRQoL, but small gains in function seem to be related to improvement. While functional gains are unlikely to change discharge destination in this frail group, they can contribute to improvements in HRQoL. These gains may be of great significance for individuals nearing the end of life and should be taken into account in resource allocation.

## Background

In keeping with the changes in the age structure of populations in many western countries, the demand and costs for health care for frail older people are anticipated to increase markedly over the next few decades [1], with important consequences from both individual and societal viewpoints. Traditionally older people entering residential care have been offered fewer therapy and health services as their health-related quality of life (HRQoL) is perceived to be poor [2] and deteriorating with little hope of reversal.

The HRQoL among older people has been reported in a variety of samples. However, the majority of studies have been based on community-dwelling older people [3-6] or on groups of people with particular health conditions [7-13]. There is a paucity of studies that have examined HRQoL in residential aged care [14], and none have reported HRQoL among people who are waiting for, or who have entered, residential aged care.

Older people caught at the interface of hospital and aged care services have historically been stigmatised as 'bed blockers' and faced lengthy waits for appropriate care [15]. In Australia, the Transition Care Program was introduced in 2005 to facilitate the transitions of older people between the acute and aged care systems. In this program, transition care is provided at the conclusion of an inpatient hospital episode and involves up to 12 weeks of support and active management. It is designed to allow older people additional time and assistance to complete the restorative process, optimise functional capacity and finalise longer term living arrangements [16].

As part of a randomized controlled trial evaluating a transitional care facility [15], we assessed the HRQoL of older people awaiting first-time transfer to residential aged care using the Assessment of Quality of Life (AQoL) measure [17-19]. We also considered the relationship between HRQoL and survival, hospital usage, residence status and function over a four-month follow-up period.

## Methods

### Participants

Participants were those who consented to be part of a trial evaluating a transitional care facility for older hospitalised patients waiting for a residential aged care bed. The study has been reported in detail elsewhere [15]. In brief, participants were recruited from the three public hospitals in the southern region of Adelaide, South Australia. Participants were recruited while they were in hospital and written consent was gained from either the participant or their carer. Participants were then randomized in a 2:1 ratio to either transfer to the transitional care facility or to receive usual care in hospital while waiting for entry to a residential care facility. Ethical approval for the study was gained from the relevant committees of the three participating hospitals.

### Measures

Questionnaires concerning function (modified Barthel Index [20]) and HRQoL (measured with the AQoL [18]) were completed at baseline and four-month follow-up. Proxy respondents completed the questionnaires when participants were unable to self-respond to the questionnaires.

The *AQoL *[17,18] was used to assess HRQoL. In the AQoL, a utility score is derived from scores on four dimensions measuring Independent living, Social relationships, Physical senses and Psychological well-being. The AQoL is scored on a life-death scale, where the lowest possible value, -0.04, represents states that are valued worse than death, 0.00 represents death-equivalent states and the highest value, 1.00, represents the best possible life state. The AQoL has been extensively validated across clinical and community settings [18]. Australian population norms show a mean AQoL of 0.75 (95%CI:0.72–0.78) for those aged 70–79 years and 0.66 (95%CI: 0.60–0.72) for those aged 80 years or more [19]. In the present study and irrespective of whether completed by self or proxy respondent, the reliability of the AQoL for study participants was Cronbach's α = 0.56, lower than that published elsewhere [19].

The *modified Barthel Index *[20] assesses function using ten items that cover mobility and self-care domains. The ten items are weighted and summed to give a modified Barthel index score between 0 and 100. A score of 0 indicates total dependence in activities of daily living, while 100 indicates complete independence in the mobility and self-care domains. The reliability of the modified Barthel Index in the present study was high (Cronbach's α = 0.96).

Participant status at four months (living in permanent care, living at home, in hospital, other place or residence, dead) and hospital usage in the four month follow-up period were also assessed. Date of death was recorded for decedents and verified from case notes and obituary listings.

### Data analysis

Because the distributions of the baseline and follow-up AQoL scores were skewed, non-parametric statistical tests were used. The mean was used to impute missing AQoL values (n = 2 at baseline and n = 1 survivor at follow-up). A Wilcoxon signed-rank test was used to compare baseline and follow-up AQoL utility scores. This analysis was repeated with follow-up AQoL scores of 0.0 (death-equivalent state) imputed for the participants who died during the follow-up period. AQoL scores at baseline for participants who self-consented versus those for whom proxy consent was given were compared using a Mann-Whitney U test. The minimal important difference of 0.06 [19] for the AQoL was used in the interpretation of results.

AQoL scores were dichotomised (≤ 0.0 vs >0.0) and Cox proportional hazards regression [21] was used to assess the effect of baseline AQoL on mortality. The effect of baseline AQoL ≤ 0.0 upon hospital utilisation and place of residence during follow-up was assessed using binary and multinomial logistic regression models respectively. A linear regression model was fitted to assess the effect of change in function upon change in AQoL among the surviving cohort. Age and gender were included as covariates in this model. All analyses were performed using SPSS, version 12.0 [22]. A significance level of α = 0.05 was used in the analyses.

## Results

A total of 320 participants were enrolled in the study. Three participants subsequently withdrew, and thus 317 participants completed baseline assessments, while 230 participants survived to four month follow-up and completed assessments at the follow-up point. As described in Crotty et al. [15], the average age of participants was 82.9 (SD 7.9) years. Half of the participants were men, as one of the participating hospitals provided prioritized health services for war veterans. The participants were frail, with a mean modified Barthel index score of 47.3 (SD 30.4) at baseline. Almost 30% of the participants were admitted to hospital with musculoskeletal problems (falls, fractures and soft tissue injuries). There were no differences in baseline measures between the participants who were allocated to the transitional care facility and the participants who were allocated to receive usual care. Proxy respondents completed the questionnaires for the majority (n = 250; 79%) of the participants.

### HRQoL among all participants

**Figure 1:**
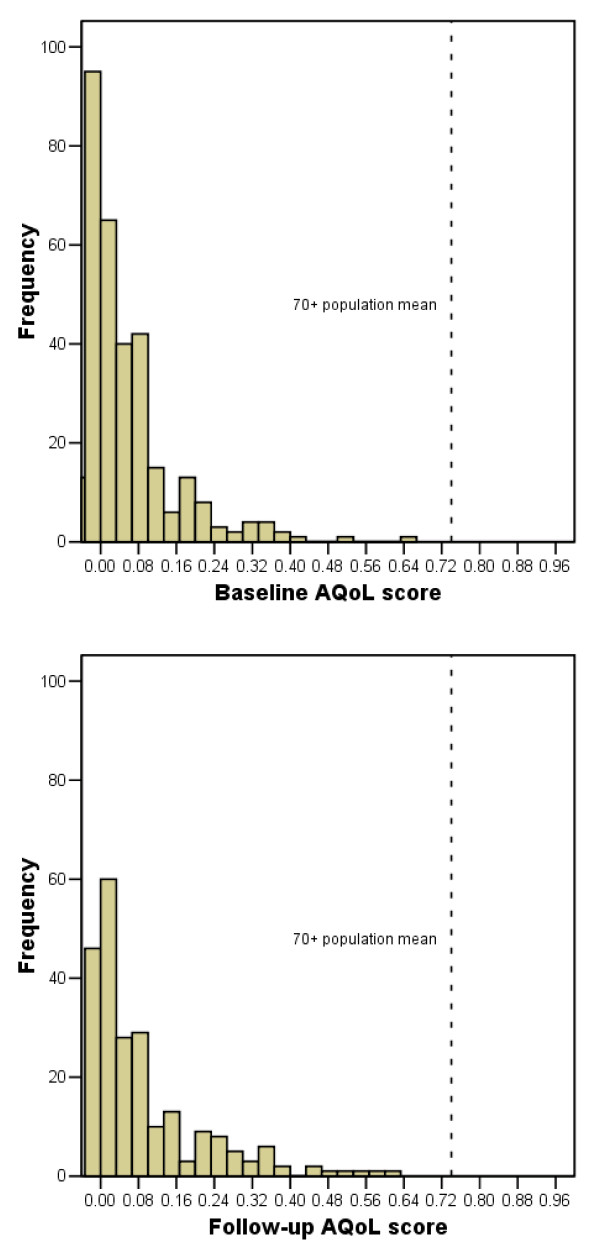
Distribution of Assessment of Quality of Life scores

The median AQoL utility scores indicated extremely poor HRQoL at baseline (median 0.02; 95%CI 0.02 – 0.04) and at follow-up (0.05; 95%CI 0.03 – 0.06). Figure 1 demonstrates that all of the baseline and follow-up AQoL scores fell below the comparable mean norm score of 0.73 for Australians aged 70 years or more [19]. Furthermore, 34% of all participants rated themselves as in a state worse than death at baseline, and 81% of the sample rated themselves at or near death-equivalent health-related quality of life (AQoL < 0.10).

As shown in Table 1, the independent living scale had the lowest baseline and follow-up median scores, suggesting poorest HRQoL in this domain. Social relationships scores were intermediate. Physical sense and psychological well-being had the highest median utility scores at both time points, contributing the least disutility to the overall AQoL score.

**Table 1:** AQoL scale summary statistics

**Variable**	**Mean**	**SD**	**95%CI**	**Median**	**Q1**^†^	**Q3**^‡^
**Baseline (n = 317)**						
AQoL utility	0.06	0.10	0.05 – 0.07	0.02	-0.01	0.08
Independent Living	0.09	0.14	0.07 – 0.10	0.00	0.00	0.09
Social relationships	0.48	0.32	0.45 – 0.52	0.31	0.25	0.79
Physical sense	0.76	0.23	0.73 – 0.78	0.84	0.63	0.94
Psychological well-being	0.78	0.21	0.76 – 0.81	0.85	0.67	0.93

**Follow-up (n = 230)**						
AQoL utility	0.09	0.13	0.07 – 0.11	0.05	0.00	0.13
Independent Living	0.11	0.17	0.09 – 0.14	0.00	0.00	0.24
Social relationships	0.55	0.30	0.51 – 0.59	0.69	0.27	0.80
Physical sense	0.79	0.16	0.77 – 0.81	0.82	0.75	0.88
Psychological well-being	0.86	0.15	0.84 – 0.88	0.90	0.83	0.93

Imputed follow-up (n = 317)*						
AQoL utility	0.06	0.11	0.05 – 0.08	0.01	0.00	0.08

* AQoL of 0.0 imputed for 87 decedents at follow-up

†, ‡ – 25th and 75th percentiles respectively

A sensitivity analysis with death-equivalent HRQoL equal to 0.0 imputed for the 87 decedents showed no significant difference between AQoL at baseline and follow-up (median improvement 0.01; 95%CI 0.00–0.01; P = 0.170).

**Figure 2:**
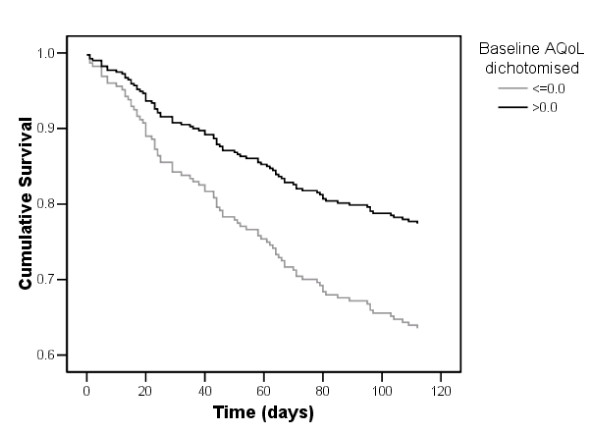
Survival function for participants with baseline AQoL≤0.0 versus  >0.0

As shown in Figure 2, baseline AQoL ≤ 0.0 was a significant predictor of mortality at four months follow-up (age and gender adjusted HR 1.7; 95%CI 1.2 – 2.7). A total of 103 participants (33%) presented or were admitted to hospital in the follow-up period.

### HRQoL among surviving participants

Amongst the subset of 230 participants who survived the four month follow-up period, the proportion who rated themselves in a state worse than death at baseline was similar (30%) to the full sample, as was the proportion who reported an AQoL < 0.10 (77%). At four month follow-up, 20% of the surviving participants rated themselves as in a state worth than death, and 71% reported AQoL utility scores of <0.10. Among the 230 surviving participants, there was a small increase in follow-up AQoL score compared with baseline AQoL (median improvement 0.01; 95%CI 0.00 – 0.03; P = 0.003). However, the effect of baseline AQoL ≤ 0.0 on hospital utilisation during follow-up (age and gender adjusted OR 0.8; 95%CI 0.5 – 1.3) was not significant. Baseline AQoL ≤ 0.0 was not a significant predictor of place of residence among the survivors (age and gender adjusted OR_home vs res care _0.6; 95%CI 0.2–1.7; age and gender adjusted OR_other vs res care_0.8; 95%CI 0.4–1.7).

Improved function, as measured by the change in the modified Barthel Index, was a statistically significant predictor of improved AQoL among the surviving cohort. A multiple linear regression model showed that every 10 unit change in the modified Barthel index predicted a 0.03 change (SD = 0.003) in the AQoL score.

### HRQoL among proxy and nonproxy respondents

Participants for whom the questionnaires were completed by proxy (n = 250) had significantly lower median AQoL scores at baseline (0.01; 95%CI 0.00 – 0.02) than for those 67 participants who self-completed the AQoL (0.07; 95%CI 0.05 – 0.09; P < 0.001). A similar result held at follow-up (proxy-completion median 0.03; 95%CI 0.02 – 0.05; self-completion 0.09; 0.08 – 0.13; P < 0.001). Among the self-completers there was a small, but not statistically significant, increase in follow-up AQoL score from baseline (median improvement 0.05; 95%CI -0.01 – 0.09; P = 0.093).

### HRQoL among usual care recipients

Among the subset of participants who received usual care (n = 105), the baseline and follow-up median AQoL scores were again very low (baseline 0.02; 95%CI 0.00 – 0.04; follow-up 0.08; 95%CI 0.04 – 0.11). The increase in AQoL score at follow-up from baseline was modest but statistically significant (median improvement 0.03; 95%CI 0.01 – 0.06; P = 0.001).

## Conclusion

Hospitalized patients waiting for entry to residential care were shown in this study to have very poor HRQoL at both baseline and follow-up. A death-equivalent or worse than death-equivalent AQoL at baseline was a significant predictor of mortality over the ensuing four months of follow-up. The study also demonstrated that an improvement in function predicted an improvement in quality of life over the follow-up period.

There was a small but statistically significant improvement in AQoL utility from baseline to follow-up among the surviving cohort. However, a sensitivity analysis in which a death-equivalent AQoL of 0.0 was imputed demonstrated no significant difference in AQoL scores between the two time points. Slight decreases in HRQoL measured by the EQ-5D at six month follow-up were recently reported in a trial of home based medication review post-hospitalisation for older people [23]. Thus while improvement in HRQoL is possible, any such gains without a very targeted approach are likely to be negligible.

The positive relationship between change in function and change in AQoL among the study participants demonstrates that improvements in function can improve HRQoL, and conversely declines in function can impact negatively on HRQoL. Sturm similarly reported a strong correlation between the Barthel index and AQoL three months post stroke [13]. In hospitals once a decision is made that a patient is moving into residential care, rehabilitation and therapy services are frequently withdrawn. The findings in the present study suggests that if rehabilitation services set realistic goals for functional improvements, then gains in function can lead to small improvements in HRQoL for very frail older people at the beginning of their stay in residential aged care.

From a health economic perspective, it could be argued that if the HRQoL among people waiting for first-time transfer to residential care is virtually equivalent to death, there is little point in intervening with health care programs that may have little health benefit and that drain economic resources from other more cost-effective interventions. Although this argument fails the rule of rescue which asserts that "it is the duty of society to assure equitable access to an adequate level of care for all" [24], it is consistent with the long history, starting with Plato, of those who have argued against the prolongation of the act of dying and who, more recently, have explored the role of clinicians in using societal resources to assist this prolongation. This dilemma – between the quality of life and the quantity of life – was first raised in the quality of life medical literature by Long in the context of the appropriate allocation of resources to those *in extremis *[25]. Although Long argued for an ethical and humane solution to this dilemma, more recently programs have focussed on the political imperative to provide care whilst reducing costs [2]. The results of this study suggest equivocal findings and that inflexible philosophical positions regarding the cessation or prolongation of care may not necessarily apply to individuals.

This extremely frail group of older people was quite heterogeneous. One third of the study sample had extremely poor HRQoL at baseline and there was a high mortality rate in the four month follow-up period amongst these participants. However, 19% of the sample did not have this very poor HRQoL at baseline (ie AQoL score > 0.1), and gains in HRQoL were apparent at follow-up among the survivors.

There are several caveats that must be borne in mind when interpreting the results reported here. First, the proxy-reported HRQoL scores were significantly lower than the self-reported scores. This finding is consistent with the literature, and is a potential threat to the study findings. Where self-report and proxy utility scores have been compared, the self-reported scores are generally higher than those of the proxies [26,27]. The implication is that self-respondents rate their HRQoL higher than do external observers, possibly due to adaptation or because proxies may be either unaware of all aspects of self-respondents' lives or may focus on the negative aspects of a person's life [28]. There is, generally, highest agreement between self and proxy assessments of more objective measures (e.g. mobility) and greater discrepancy in the subjective areas of life (e.g. social relationships) [29], although not all studies have reported this [26,27]. Finally, while the sampling frame in the present study represented the population of hospital patients awaiting residential aged care, the generalisability of our findings to other localities with a different climate of health and aged care service provision remains unknown.

The difficulty posed in studies of frail older persons is in judging when proxy reports should be used. Self-report assumes that the individual has the necessary insight into his or her own life to be able to provide meaningful assessments. In the present study, the capacity to self-complete the study questionnaires was ascertained at baseline when consent was granted either by the participant or their proxy. Although we acknowledge the limitations of mixing proxy with self-reported responses, on balance we adopted the position that it was better to include proxy reports for those unable to self-complete our questionnaire than to exclude them from the study altogether, which would lead to a biased sample.

Second, because of the high mortality rate in the study sample, we were limited to a reasonably short follow-up period. Another measure of AQoL at 12 months would give further insight as to whether improvements in function can improve HRQoL and/or survival time. To minimize respondent burden a very constrained number of outcome measures were used. Additional outcome measures, including an objective assessment of function such as the Timed-Up-and-Go [30], would have helped to further elucidate the relationship between function and HRQoL. Finally, questionnaire based outcome assessment may not fully reflect each individual's HRQoL and qualitative interviews may have revealed "response shift" where a person re-evaluates themselves after an intervention as better or worse than at the beginning [31]. The effects of such changes following hospitalization in frail older people have not been explored and may be important in augmenting our understanding of this group.

There are two main policy implications from the present study, subject to replication of the study findings in other studies. First, the findings suggest that whilst the patient is in hospital planned rehabilitation and therapy care should be continued in the hospital setting. Second, the results suggest the need for a high level of coordination of care during the transition period from hospital to residential care. The Transition Care Program [16,32] recently introduced as a joint initiative of the federal and state Australian governments may offer improved coordination of the move from hospital to residential care, as may subacute hospital geriatric assessment and rehabilitation services [33]. The evidence base for the latter's efficacy in improving patients' functional status and reducing hospital discharge rates to residential care, along with the uncertainty that out-of-hospital programs offer an effective alternative, has led to a recent call to prioritise redressing the inadequate provision of these services in some regions ahead of Transition Care Programs [34].

The denial of rehabilitation to elderly people from nursing homes (the "lost tribe") has been described as inappropriate [2]. Policy makers and clinicians need to consider the implications of this study for health resource allocation, and recognize that small improvements in HRQoL may be of great significance for individuals nearing the end of life.

## List of abbreviations

HRQoL: Health related Quality of Life; AQoL: Assessment of Quality of Lifel; CI: Confidence Interval; OR: Odds Ratio

## Competing interests

The authors declare that they have no competing interests.

## Authors' contributions

LG participated in study design and coordination, performed the statistical analyses and drafted the manuscript. GH participated in the statistical analyses and helped to draft the manuscript. MC conceived of the study, participated in its design and coordination and helped to draft the manuscript. All authors read and approved the final manuscript.

## References

[B1] Madge A (2000). Long-term aged care: Expenditure trends and projections. Canberra.

[B2] Stott DJ, Langhorne P, Knight PV (2008). Multidisciplinary care for elderly people in the community. Lancet.

[B3] Osborne RH, Hawthorne G, Lew EA, Gray LC (2003). Quality of life assessment in the community-dwelling elderly: validation of the Assessment of Quality of Life (AQoL) Instrument and comparison with the SF-36. Journal of Clinical Epidemiology.

[B4] Power M, Quinn K, Schmidt S (2005). Development of the WHOQoL-OLD module. Quality of Life Research.

[B5] Walters SJ, Munro JF, Brazier JE (2001). Using the SF-36 with older adults: a cross-sectional community-based survey. Age and Ageing.

[B6] Holland R, Smith RD, Harvey I, Swift L, Lenaghan E (2004). Assessing quality of life in the elderly: a direct comparison of the EQ-5D and AQoL. Health Economics.

[B7] Dugan E, Cohen SJ, Robinson D, Anderson R, Preisser J, Suggs P (1998). The quality of life of older adults with urinary incontinence: determining generic and condition-specific predictors. Quality of Life Research.

[B8] Logsdon RG, Gibbons LE, McCurry SM, Teri L (2002). Assessing quality of life in older adults with cognitive impairment. Psychosomatic Medicine.

[B9] Naumann VJ, Byrne GJA (2004). WHQoL-Bref as a measure of quality of life in older patients with depression. International Psychogeriatrics.

[B10] Schrag A, Jahanshahi M, Quinn N (2000). How does Parkinson's disease affect quality of life? A comparison with quality of life in the general population. Moving Disorders.

[B11] Francis HW, Chee N, Yeagle J, Cheng A, Niparko JK (2002). Impact of cochlear implants on the functional health status of older adults. Laryngoscope.

[B12] Harris A, Gospodarevskaya E, Callaghan J, Story I (2001). The cost effectiveness of a pharmacist reviewing medication among the elderly in the community. Australasian Journal on Ageing.

[B13] Sturm JW, Osborne RH, Dewey HM, Donnan GA, Macdonell RAL, Thrift AG (2002). Brief comprehensive quality of life assessment after stroke: The Assessment of Quality of Life instrument in the North East Melbourne Stroke Incident Study (NEMESIS). Stroke.

[B14] Kane RA, Kling KC, Bershadsky B, Kane RL, Giles K, Degenholtz HB (2003). Quality of life measures for nursing home residents. Journal of Gerontology: Medical Sciences.

[B15] Crotty M, Whitehead C, Wundke R, Giles LC, Ben-Tovim D, Phillips PA (2005). A transitional care facility for hospitalised older adults awaiting a nursing home bed: a randomised controlled trial. British Medical Journal.

[B16] Australian Government Department of health and Ageing (2005). The transition care program guidelines.

[B17] Hawthorne G, Richardson J, Day NA (2001). A comparison of the Assessment of Quality of Life (AQoL) with four other generic utility instruments. Annals of Medicine.

[B18] Hawthorne G, Richardson J, Osborne R (1999). The Assessment of Quality of Life (AQoL) instrument: a psychometric measure of health-related quality of life. Quality of Life Research.

[B19] Hawthorne G, Osborne R (2005). Population norms and meaningful differences for the Assessment of Quality of Life (AQoL) measure. Australian and New Zealand Journal of Public Health.

[B20] Shah S, Vanclay F, Cooper B (1989). Predicting discharge status at commencement of stroke rehabilitation. Stroke.

[B21] Cox DR (1972). Regression models and life-tables (with discussion). Journal of the Royal Statistical Society, Series B.

[B22] SPSS Inc (2004). SPSS for Windows v 12.0. 10.0.

[B23] Holland R, Lenaghan E, Harvey I, Smith R, Shepstone L, Lipp A (2005). Does home based medication review keep older people out of hospital? The HOMER randomised controlled trial. British Medical Journal.

[B24] Jonsen AR (1986). Bentham in a box: Technology assessment and health care allocation. Law, Medicine and Health Care.

[B25] Long PH (1960). On the quality and quantity of life. Medical Times.

[B26] Naglie G, Tomlinson G, Tansey C, Irvine J, Ritvo P, Black S (2006). Utility-based quality of life measures in Alzheimer's disease. Quality of Life Research.

[B27] Wlodarczyk JH, Brodaty H, Hawthorne G (2004). The relationship between quality of life, Mini-Mental State Examination, and the Instrumental Activities of Daily Living in patients with Alzheimer's disease. Archives of Gerontology and Geriatrics.

[B28] Cummins RA (2002). Proxy responding for subjective well-being: A review. International Review of Research in Mental Retardation.

[B29] Herrman H, Hawthorne G, Thomas R (2002). Quality of life assessment in people living with psychosis. Social Psychiatry and Psychiatric Epidemiology.

[B30] Podsiadlo D, Richardson S (1991). The Timed "Up & Go": A test of basic functional mobility for frail elderly persons. Journal of the American Geriatrics Society.

[B31] Sprangers MA, Schwartz CE (1999). Integrating response shift into health-related quality of life research: A theoretical perspective. Social Science and Medicine.

[B32] Australian Government Department of Health and Ageing (2008). National Evaluation of the Transition Care Program. Canberra.

[B33] Gray L, Moore K, Smith R, Dorevitch M (2007). Supply of inpatient geriatric medical services in Australia. Internal Medicine Journal.

[B34] Gray LC, Travers CM, Bartlett HP, Crotty M, Cameron ID (2008). Transition Care: Will it deliver?. Medical Journal of Australia.

